# Treatment of Potential Postpartum Eclampsia in a Python Using Traditional Chinese Veterinary Medicine: A Case Report

**DOI:** 10.1002/vms3.71122

**Published:** 2026-07-29

**Authors:** Shuo Yang, Yuting Liu, Jie Mi, Li‐Jen Chang, Wu‐Ren Ma

**Affiliations:** ^1^ College of Veterinary Medicine Northwest A&F University Yangling People's Republic of China; ^2^ Xi'an Veterinary Teaching Hospital Northwest A&F University Xi'an People's Republic of China; ^3^ Department of Small Animal Clinical Sciences Virginia Maryland College of Veterinary Medicine Blacksburg Virginia USA; ^4^ Institute of Traditional Chinese Veterinary Medicine Northwest A&F University Yangling People's Republic of China

**Keywords:** eclampsia, python, traditional chinese veterinary medicine, treatment, *Yin* and blood deficiency

## Abstract

According to the traditional Chinese veterinary medicine (TCVM) theory, *Yin* deficiency contributes to the acute onset of eclampsia. This condition arises primarily from the substantial loss of blood and nutrients to the foetus during pregnancy, leading to insufficient nutrition and blood supply. Consequently, the muscles and veins are deprived of essential nutrients, which manifests as convulsive symptoms. This case report presents a case in which the TCVM principles of eclampsia treatment were applied to manage seizures‐like signs in a python with postpartum *Qi* and blood deficiency. Following treatment with Chinese herbs, the python regained normal mentation, appearance and defecation. These findings suggest that postpartum *Qi* and blood deficiency in pythons can be effectively treated with Chinese herbs guided by the TCVM theory, providing a valuable reference for addressing similar conditions in reptiles.

## Graphical abstract

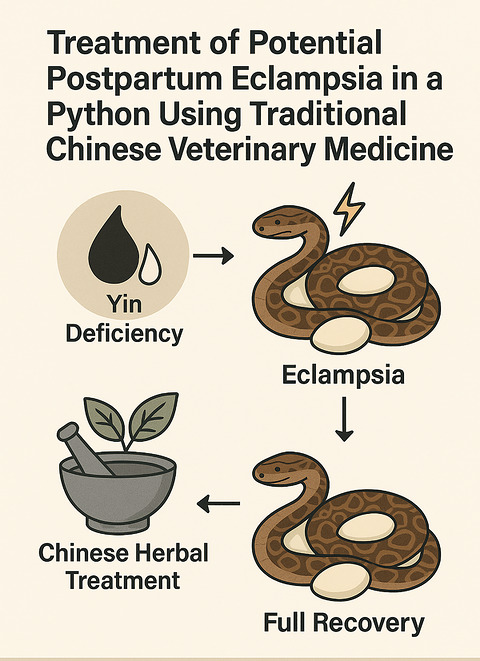

## 1 Introduction

Golden python is an albino or yellow variant of the Burmese python (*Python bivittatus*), which belongs to Pythonidae. Pythonidaes are restricted to the old world and occupy diverse habitats in Africa, Asia and Australia (Miller and Fowler 2014). Burmese python is one of the largest species of snakes which is native to a large area of Southeast Asia and listed as vulnerable on the IUCN Red List (Stuart et al. 2019).

In traditional Chinese medicine (TCM), eclampsia is referred to as pregnancy epilepsy syndrome. The primary symptoms include dizziness, headaches, sudden fainting, upward gazing, clenched teeth, limb twitching and opisthotonos (arching of the back), which typically occur during the late stages of pregnancy, labour or shortly after delivery. Patients may briefly regain consciousness, but the condition can recur upon waking or, in severe cases, lead to prolonged unconsciousness (Qi et al. 2020). In Western medicine, eclampsia is classified as a hypertensive disorder of pregnancy, characterised by tonic–clonic seizures or coma in the absence of other causative conditions. These episodes may occur before, during or after delivery (Ford et al. 2022). TCM does not explicitly document ‘hypertensive disorders during pregnancy.’ Based on clinical manifestations, modern Chinese medicine often categorizes this condition into syndromes such as ‘pregnancy edema,’ ‘pregnancy dizziness’ and ‘pregnancy epilepsy’. In traditional Chinese veterinary medicine (TCVM), eclampsia occurs when the tendons and meridians are not adequately nourished. During eclampsia, tissues cannot obtain adequate nutrients, resulting in more severe clinical signs. According to this theory, nutrients are stored to support foetal development, leading to potential malnutrition and subsequent eclampsia during pregnancy (Zhang et al. 2006). In this study, Chinese herbs were administered to a python with potential eclampsia diagnosed using TCVM theory, with favourable therapeutic outcomes. The findings of this case report provide a valuable reference for the treatment of pythons with similar symptoms.

## 2 Signalment

**FIGURE 1:**
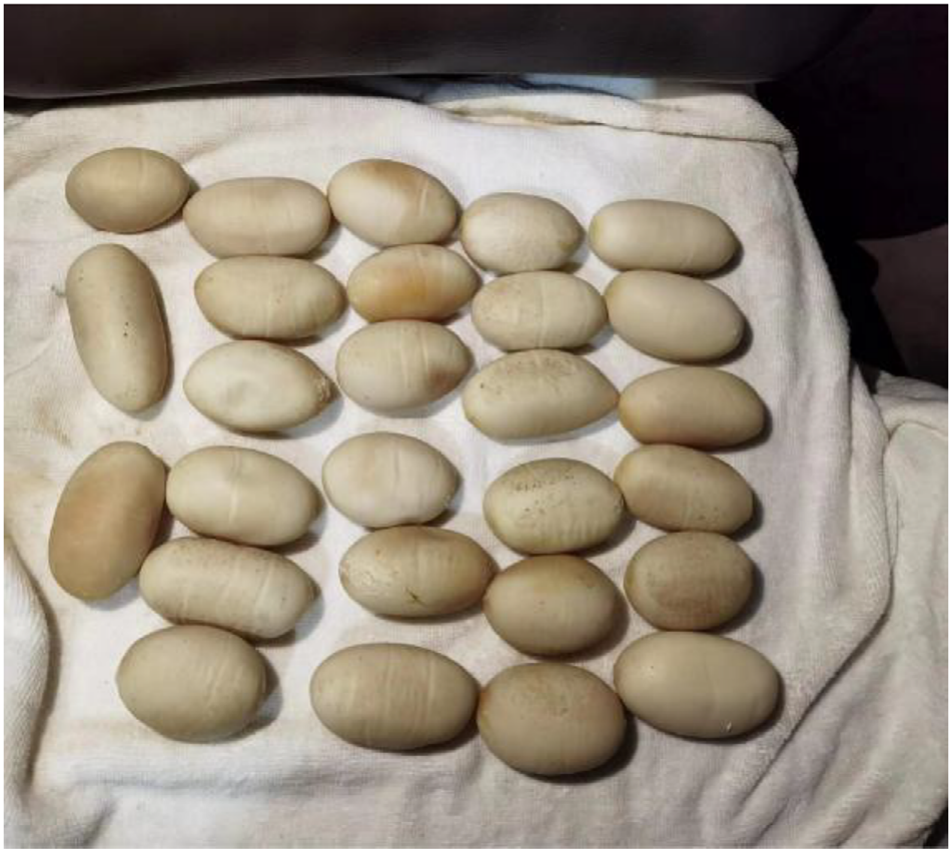
The appearance of eggs laid by the snake.

On 14 April 2021, a female golden python weighing 20 kg and of unknown age laid more than 20 irregularly shaped eggs (Figure 1). The python was kept in an artificially temperature‐controlled enclosure in a reptile and amphibian exhibition facility. The overall environment was designed to simulate a tropical rainforest or humid woodland habitat. The bottom of the enclosure was covered with moisture‐retaining and cushioning substrates, such as coconut coir, bark, humus and fallen leaves. Branches, rocks, hiding shelters, artificial plants and a large water basin were also provided, allowing the python to coil, hide, move around and soak. The daytime ambient temperature was generally maintained at approximately 28°C–30°C, with a warm area of about 32°C–35°C and a cool area of about 24°C–27°C. The night‐time temperature was kept above 24°C, and the relative humidity was maintained at 60%–75%. Two weeks prior to oviposition, muscle twitching was noticed. The owner considered the twitching to be mild and potentially associated with normal incubation activity; therefore, veterinary medical advice was not taken immediately. By mid‐May 2021, the clinical signs were even worse, with paroxysmal generalised tremors, convulsions, and rigidity occurring throughout the body, and no defecation had been observed for three months. On 7 June 2021, the python was presented at the Veterinary Teaching Hospital of the Northwest Agriculture and Forestry University for treatment.

## 3 Clinical Findings

**FIGURE 2:**
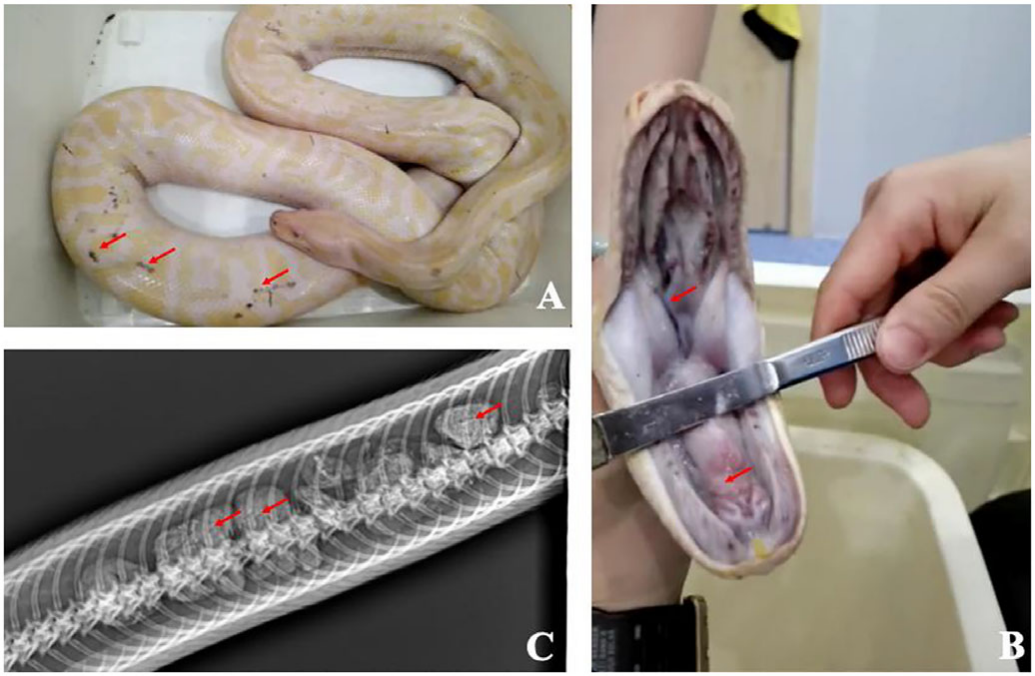
Examination findings of the patient. (A) Appearance of the body surface during the first visit, multiple subcutaneous haemorrhagic spots were visible (indicated by red arrows). (B) Pallor of the oral mucosa, ulceration at the tip of the tongue and blood stasis were observed in the oral cavity (indicated by red arrows). (C) Radiographic imaging of the patient, indicating the presence of soft tissue opacity materials, which was considered an accumulation of dry faecal matter according to the history and physical examination (indicated by red arrows).

Clinical examination revealed a dull mentation, marked lethargy, deterioration and subcutaneous haemorrhage (Figure 2A). Physical examination revealed pallor of the oral mucosa, ulceration at the tip of the tongue, blood stasis in the oral cavity (Figure 2B) and a palpable solid mass in the posterior abdominal region. Radiology reports revealed the accumulation of soft tissue opacity, which was considered to be dry faecal accumulation based on the anatomy of python and clinical signs (Figure 2C).

## 4 TCVM Diagnosis

Based on the patient's history and physical examination, the patient was diagnosed with *Yin* deficiency, insufficient production of bodily fluids and intestinal dryness, which collectively contributed to constipation. Furthermore, the inability of blood to adequately nourish the muscles and veins precipitates potential seizure episodes.

## 5 Treatment and Outcome

**FIGURE 3:**
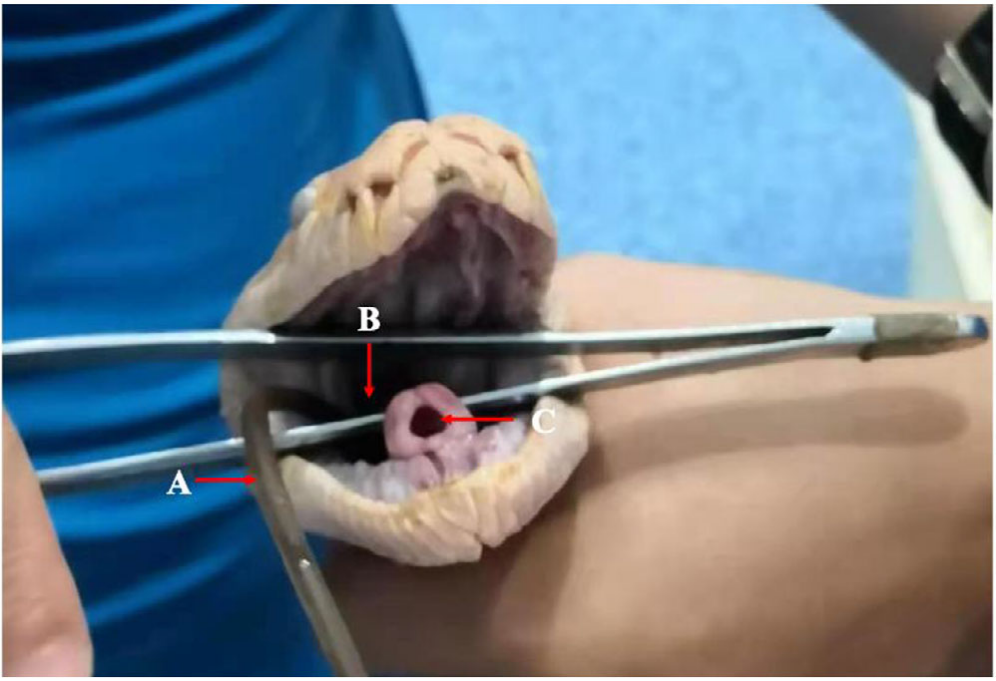
Administration of Chinese herbal medicines. (A) Feeding tube with Chinese herbs. (B) The oesophagus and (C) glottis.

**FIGURE 4:**
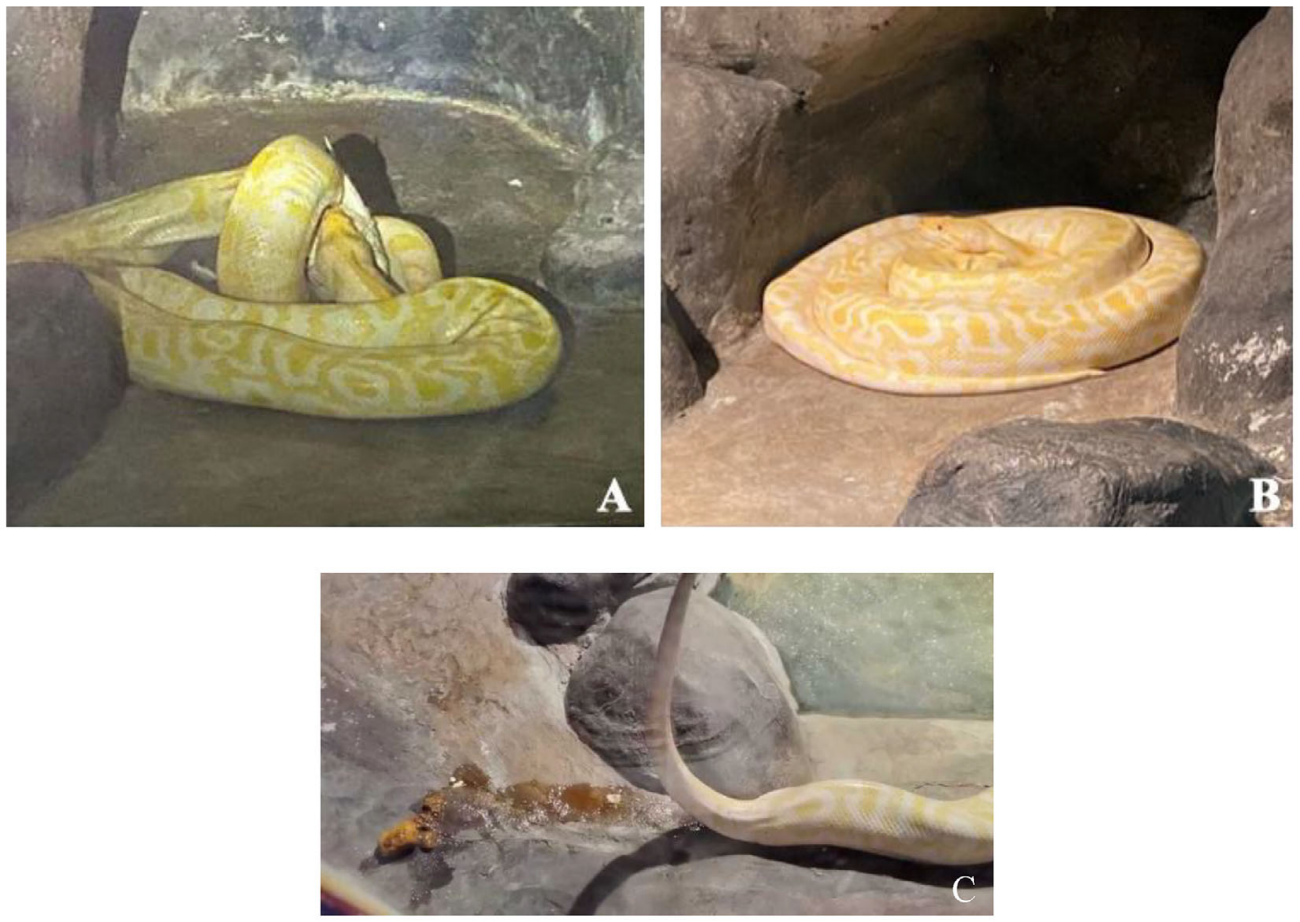
Appearance of the patient after treatment. (A) The patient was eating. (B) The skin of the patient had returned to normal; she exposed her head outside the body, and her eyes were bright. (C) Defecation returned to normal.

The principle of the treatment was to tonify *Yin*, moisten the intestines, promote bowel movements, replenish *Qi* and blood, strengthen muscles and tendons, and control epilepsy. The patient was administered two formulations of Chinese herbs in extract form, which were mixed and ground into a fine powder. The preparation process involved measuring the weight of the extracts corresponding to the weight of the herbal slices in the formulations and mixing and grinding them into a fine powder with the dose of 0.1 g/kg of body weight. The powder was dissolved in warm water and the solution was administered from a gastric gavage (Figure 3). The duration of herbal treatment was 36 days, from 9 June 2021 to 14July 2021. The first formulation was prescribed for 14 days from 9 June 2021 to 22 June 2021, followed by a follow‐up visit and the second formulation was prescribed for 22 days from 23 June 2021 to 14 July 2021. The first formulation included 30 g of *Xuan Shen* (*Scrophularia*, 9 g of extract), 24 g of *Mai Men Dong* (*Ophiopogon*, 7.2 g of extract), 24 g of *Sheng Di Huang* (*Rehmannia*, 7.2 g of extract), 9 g of *Da Huang* (*Rheum*, 3 g of extract), 4.5 g of *Mang Xiao* (*Mirabilitum*, 1.8 g of extract), 10 g of *Xing Ren* (*Armeniaca*, 1 g of extract), 12 g of *Jie Geng* (*Platycodon*, 3 g of extract), 12 g of *Ban Xia* (*Pinellia*, 1 g of extract) and 6 g of *Xi Xin* (*Asarum*, 1 g of extract), the functions of which are detailed in Table 1. After 2 weeks of treatment, seizure‐like symptoms were observed, but the patient remained inappetent. A second herbal formulation was introduced on June 23, at the same dosage and frequency as the first prescription (Table 2). The second formulation included: 12 g of *Dan Shen* (*Salvia*, 2.4 g of extract), 6 g of *Tao Ren* (*Persica*, 0.6 g of extract), 10 g of *Huang Qi* (*Astragalus*, 1.5 g of extract), 10 g of *Dang Gui* (*Angelica*, 4 g of extract), 10 g of *Chuan Xiong* (*Ligusticum*, 3.4 g of extract), 10 g of *Fu Ling* (*Poria*, 1 g of extract), 10 g of *Ren Shen* (*Ginseng*, 2.5 g of extract), 10 g of *Bai Zhu* (*Atractylodes*, 3 g of extract) and 9 g of *Gan Cao* (*Glycyrrhiza*, 1.5 g of extract), followed by 6 g of *San Qi* (*Notoginseng*, 3 g of extract), 6 g of *Chen Pi* (*Citrus*, 1 g of extract), and 6 g of *Ji Xue Teng* (*Millettia*, 0.4 g of extract). By 14 July, the owner observed a significant improvement in skin colour (less subcutaneous petechiae was observed), and the python became brighter, more alert and more responsive with normal defecation after receiving TCVM treatment (Figure 4A–C).

**TABLE 1:** The Chinese herbal formula used in the first prescription (Huisheng and Preast 2010).

Pin Yin name (Common English name)	Actions
*Xuan Shen* *(Scrophularia)*	Clear heat and cool blood Nourish *Yin* and release toxins Soften hardness and dissipate nodules
*Mai Men Dong* *(Ophiopogon)*	Moisten lungs, nourish *Yin* Nourish stomach *Yin* and generate bodily fluids Eliminate heart heat and irritability Moisten intestines and promote bowel movements
*Sheng Di Huang* *(Rehmannia)*	Clear heat and cool blood Nourish *Yin* and generate bodily fluids Nourish kidney *Jing* and tonify blood
*Da Huang* *(Rheum)*	Remove accumulations and guide out stagnation Drain fire and cool blood Invigorate blood and eliminate blood stagnation Regulate gallbladder and reduce jaundice
*Mang Xiao* *(Mirabilitum)*	Purge faeces downward Soften hardness Clear heat
*Xing Ren* *(Armeniaca)*	Stop cough and relieve asthma Moisten the intestines to move faeces
*Jie Geng* *(Platycodon)*	Open (disperse) the lung *Qi* Transform phlegm and expel pus Direct other herbs to upper regions of the body
*Ban Xia* *(Pinellia)*	Dry dampness and transform phlegm Descend rebellious *Qi* and stop vomiting or nausea Eliminate nodules and dissipate masses Topical application: dispel swelling and relieve pain
*Xi Xin* *(Asarum)*	Clear the surface and dispel cold Dispel wind and stop pain Warm the lungs and resolve phlegm

**TABLE 2:** The Chinese herbal formula used in the second prescription (Huisheng and Preast 2010).

Pin Yin name (Common English name)	Actions
*Dan Shen* *(Salvia)*	Invigorate blood and resolve stagnation Cool blood and eliminate abscesses Nourish blood and calm Shen
*Tao Ren* *(Persica)*	Invigorate blood and dispel stagnation Moisten the intestines and purge stool Stop asthma and cough
*Huang Qi* *(Astragalus)*	Replenish *Qi* and raise *Yang* to strengthen the spleen Tonify *Wei Qi*, stabilize the exterior, and stop sweating Clear toxins and pus, promote healing Circulate water to reduce edema
*Dang Gui* *(Angelica)*	Nourish and replenish blood Invigorate blood, stop pain Moisten intestines Regulate female reproductive organs
*Chuan Xiong* *(Ligusticum)*	Invigorate blood and move *Qi* Expel wind and stop pain
*Fu Ling* *(Poria)*	Promote urination and drain Damp Strengthen the spleen and tonify the middle burner (Zhong Jiao) Calm the Shen Transform phlegm
*Ren Shen* *(Ginseng)*	Replenish *Qi*, rescue Yuan *Qi* collapse, and strengthen Yang Tonify the spleen, lung, and heart qi Promote fluid production and relieve thirst Calm Shen
*Bai Zhu* *(Atractylodes)*	Replenish spleen *Qi* Resolve spleen dampness and promote water circulation Calm the foetus Stabilize the Exterior and stop sweating
*Gan Cao* *(Glycyrrhiza)*	Tonify the spleen and *Qi* Moisten the lungs and stop coughing Moderate spasms and relieve pain Regulate and harmonize herbs Clear heat and relieve toxicities
*San Qi* *(Notoginseng)*	Stop bleeding and relieve stagnation Invigorate blood, stop pain, and reduce swelling
*Chen Pi* *(Citrus)*	Regulate *Qi* and strengthen the spleen Dry dampness and transform phlegm
*Ji Xue Teng* *(Millettia)*	Invigorate and tonify the blood Relax the tendons and invigorate the channels

## 6 Discussion

In this case, the chief complaints were python‐laid eggs with irregular eggshell intensities and oral mucosa pallor. After TCVM diagnosis, blood stasis was observed, with clinical signs of subcutaneous haemorrhage, constipation and potential seizure episodes. During pregnancy, pythons may require more nutrients than normal to support their foetal development. However, insufficient nutrition and kidney *Yin* deficiency, exacerbated by hibernation during winter, result in eggs with irregular sizes and rough surfaces. Post‐oviposition, the python was lethargic. Furthermore, the depletion of essence and blood during pregnancy was not replenished, leading to more severe clinical signs. Nonetheless, the clinical signs observed in this case indicated *Yin* deficiency according to the TCVM. Subcutaneous petechiae may indicate impaired splenic function, resulting in inadequate blood and fluid circulation. The python's blood and bodily fluid deficiency could be the major cause of constipation, which is known as intestinal dryness in TCVM. The first formulation, *Zeng Ye Cheng Qi* Tang, was administered to nourish the *Yin*, increase fluids, relieve heat and promote intestinal movement. *Xing ren* was used to improve bowel movements based on the TCVM theory. The administration of *Jie geng* and *Xi xin* promotes lung *Qi* to further improve bowel movement because the lungs and large intestine are interconnected according to TCVM physiology. In addition, *Banxia* has been used to clear nodules, dissipate lumps and facilitate the efficient excretion of faeces (Zhou and Xu 2015).

Following the initial treatment, the clinical signs of twitching were alleviated; however, the python remained lethargic. Radiography revealed that the soft tissue opacity in the colon remained unresolved. According to TCVM physiology, loss of appetite is primarily attributed to impaired spleen function, which hinders the transportation of nutrients and leads to deficiencies in *Qi* and Blood. To address this, *Sijunzi* Tang, *Ren shen*, *Bai zhu*, *Fu ling* and *Gan cao* have been administered to nourish *Qi* and invigorate the spleen (Cai et al. 2023), thereby promoting the transformation of *Qi* and blood. In addition, *Huang Qi* was used for its efficacy in replenishing *Qi*. *Dan shen*, *Tao ren*, *Dang gui*, *Chuan xiong*, *San qi* and *Ji xue teng* were added to *Sijunzi* Tang to nourish the blood and relieve convulsions. At the same time, these drugs can also activate the biochemistry of *Qi* and blood, nourish the muscles, and alleviate the occurrence of convulsive symptoms (Wei and Jin 2026).

In TCVM, treatment is based on a holistic approach and pattern differentiation. Based on the clinical symptoms of hypertensive disorders of pregnancy, it is classified within the scope of TCM's ‘pregnancy epilepsy.’ TCM attributes the etiology of eclampsia to *Yin* deficiency, *Yang* hyperactivity, internal stirring of the liver wind and phlegm–fire disturbance (Jiao 2010). In addition, the causes of eclampsia are summarized in ‘*Shen shi nv ke ji yao* (Shen's Gynaecology Collection)’ as follows: “The first is *Yin* deficiency, the second is *Qi* stagnation, and the third is phlegm retention” (Li et al. 2016). Treatment principles often include nourishing *Yin* and blood, promoting blood circulation, resolving stasis, tonifying *Qi*, strengthening the spleen and clearing heat to restore consciousness (Jiang et al. 2020; Liu et al. 2022). This case primarily involved convulsions caused by *Y*in deficiency. The treatment principle focuses on nourishing *Y*in and blood and relieving spasms. The herbal formula Yiqi Yangyin decoction has been shown to improve serological indicators, reduce blood pressure, and achieve favourable clinical outcomes in patients with hypertensive disorders of pregnancy (Qin et al. 2024). Studies have demonstrated that combining *Danshen* and Chuanxiongqin injections for the treatment of severe preeclampsia can optimize circadian blood pressure rhythms, improve liver and kidney function, restore microenvironmental balance, and ensure maternal and foetal safety (Meng et al. 2016). Huang et al. found that *Qiju Dihuang* decoction was highly effective in treating eclampsia, lowering blood pressure, improving renal function and alleviating clinical symptoms (Huang and Lin 2019). Wu et al. reported that the use of *Sijunzi* decoction to treat pregnant women with spleen deficiency effectively reduced the incidence of hypertensive disorders of pregnancy (Wu et al. 2012).

In summary, the potential postpartum eclampsia symptoms are a result of *Yin* and blood deficiency, and prompt treatment with *Yin*‐tonifying and blood‐nourishing medications is of great importance for reptile patients in the perinatal period.

## Author Contributions

**Shuo Yang**: writing – original draft preparation. **Yuting Liu**: writing – original draft preparation. **Jie Mi**: resources. **Li‐Jen Chang**: writing – review and editing, visualization. **Wu‐Ren Ma**: conceptualization, methodology, supervision.

## Funding

The authors have nothing to report.

## Ethics Statement

The authors confirm that the ethical policies of the journal have been adhered to, as noted on the journal's Author Guidelines page. No ethical approval was required for this clinical case report with no original research data. The owner provided consent for the use of the clinical information.

## Conflicts of Interest

The authors declare no conflicts of interest.

## Data Availability

The data that support the findings of this study are available on request from the corresponding author. The data are not publicly available due to privacy or ethical restrictions.

## References

[vms371122-bib-0001] Cai, K. , Q. Zheng , S. Wei , M. Wu , H. Xiong , and H. Zhao . 2023. “Research Progress of Sijunzi Decoction and Its Predictive Analysis of Quality Markers.” Chinese Archivers of Traditional Chinese 41(11): 161–168. 10.13193/j.issn.1673-7717.2023.11.032.

[vms371122-bib-0002] Ford, N. D. , S. Cox , J. Y. Ko , et al. 2022. “Hypertensive Disorders in Pregnancy and Mortality at Delivery Hospitalization—United States, 2017–2019.” MMWR Morbidity and Mortality Weekly Report 71(17): 585–591. 10.15585/mmwr.mm7117a1. 35482575 PMC9098235

[vms371122-bib-0003] Huang, N. , and Z. Lin . 2019. “Effect of Qiju Dihuang Decoction Combined With Western Medicine in Treatment of Gestational Hypertension.” Chinese Archives of Traditional Chinese Medicine 37(7): 1734–1736. 10.13193/j.issn.1673-7717.2019.07.048.

[vms371122-bib-0004] Huisheng, X. , and V. Preast . 2010. Xie's Chinese Veterinary Herbology. Wiley‐Blackwell.

[vms371122-bib-0005] Jiang, D. , S. Yang , and J. Han . 2020. “Study on the Origin of Eclampsia.” Journal of Emergency in Traditional Chinese Medicine 29(7): 1280–1282. 10.3969/j.issn.1004-745X.2020.07.045.

[vms371122-bib-0006] Jiao, L. 2010. “Men Chengfu's Experience in Treating Pregnancy Induced Hypertension Syndrome.” Journal of Traditional Chinese Medicine 51(3): 209–210. 10.13288/j.11-2166/r.2010.03.028.

[vms371122-bib-0007] Li, J. , W. Jiang , H. Li , and Z. Fang . 2016. “Research Progress of Traditional Chinese Medicine Treatment of Hypertensive Disorders During Pregnancy.” Liaoning Journal of Traditional Chinese Medicine 43(5): 1110–1112. 10.13192/j.issn.1000-1719.2016.05.071.

[vms371122-bib-0008] Liu, D. , Z. Zhuang , S. Guo , S. Chen , B. Yao , and J. Shi . 2022. “Research Progress on Pathogenesis and TCM Prevention and Treatment of Pre‐Eclampsia.” Journal of Guangdong Pharmaceutical University 38(2): 131–137. 10.16809/j.cnki.2096-3653.2021120704.

[vms371122-bib-0009] Meng, H. , P. Zhou , Y. Zhu , and G. A. O. Xue‐mei . 2016. “Effects of TCM Therapy of Danshen Injection Combining With Ligustrazine Injection in Improving Severity of Severe Preeclampsia.” Journal of Hainan Medical University 22(4): 357–360. 10.13210/j.cnki.jhmu.20151123.009.

[vms371122-bib-0010] Miller, R. E. , and M. E. Fowler , eds. 2014. Fowler's Zoo and Wild Animal Medicine, Volume 8. Elsevier Health Sciences.

[vms371122-bib-0011] Qi, L. , H. Li , L. Wang , et al. 2020. “Effect of Compound Danshen Injection Conbined With Labetalol on Liver Function and Placental Growth Factor in Patients With Eclampsia.” Journal of Hainan Medical University 26(5): 363–366. 10.13210/j.cnki.jhmu.20200213.004.

[vms371122-bib-0012] Qin, M. , C. Xiao , Y. Wei , and B. Yao . 2024. “Meta‐Analysis of Therapeutic Effect of Yiqi Yangyin Decoction on Gestational Hypertension.” Pharmacology and Clinics of Chinese Materia Medica 40(12): 86–90. 10.13412/j.cnki.zyyl.20240423.008.

[vms371122-bib-0013] Stuart, B. , T. Q. Nguyen , N. Thy , et al. 2019. “ *Python bivittatus* (Errata Version of 2012 Assessment).” IUCN Red List of Threatened Species 2012: e.T192161A15124290.

[vms371122-bib-0014] Wei, L. , and W. Jin . 2026. “Chinese Medical Master Chen Shaohong's Experience in the Treatment of Acute Myocardical Infarction by Supplementing Qi and Activating Blood Circulation.” Liaoning Journal of Traditional Chinese Medicine 53(4): 31–34. 10.13192/j.issn.1000-1719.2026.04.007.

[vms371122-bib-0015] Wu, Y. , A. Huang , and X. Pan . 2012. “The Clinical Research of Sijunzi Decoction for Pregnancy Prevention and Control of the Spleen Deficiency Physical Intervention With Hypertension.” China Medicine and Pharmacy 2(15): 85–86.

[vms371122-bib-0016] Zhang, J. , T. X. Wu , and G. J. Liu . 2006. “Chinese Herbal Medicine for the Treatment of Pre‐Eclampsia.” Cochrane Database of Systematic Reviews 2006: CD005126. 10.1002/14651858.CD005126.pub2. 16625625 PMC8865522

[vms371122-bib-0017] Zhou, Q. , Z. Xu , and X. Zhou . 2015. “Exploration on the Treatment of Constipation With Zengye Decoction and Zengye Chengqi Decoction Based on Syndrome Differentiation.” Liaoning Journal of Traditional Chinese Medicine 42(11): 2108–2110. 10.13192/j.issn.1000-1719.2015.11.020.

